# An Insulin‐Inspired Supramolecular Hydrogel for Prevention of Type 1 Diabetes

**DOI:** 10.1002/advs.202003599

**Published:** 2021-04-09

**Authors:** Mohan Liu, Zhongyan Wang, Dandan Feng, Yuna Shang, Xinxin Li, Jianfeng Liu, Chen Li, Zhimou Yang

**Affiliations:** ^1^ Tianjin Key Laboratory of Biomedical Materials Biomedical Barriers Research Centre Institute of Biomedical Engineering Chinese Academy of Medical Sciences & Peking Union Medical College Tianjin 300192 P. R. China; ^2^ Key Laboratory of Bioactive Materials Ministry of Education College of Life Sciences State Key Laboratory of Medicinal Chemical Biology Collaborative Innovation Centre of Chemical Science and Engineering and National Institute of Functional Materials Nankai University Tianjin 300071 P. R. China; ^3^ Tianjin Key Laboratory of Radiation Medicine and Molecular Nuclear Medicine Institute of Radiation Medicine Chinese Academy of Medical Sciences & Peking Union Medical College Tianjin 300192 P. R. China; ^4^ Jiangsu Center for the Collaboration and Innovation of Cancer Biotherapy Cancer Institute Xuzhou Medical University Xuzhou Jiangsu 221004 P. R. China

**Keywords:** autoimmunity, immunoregulation, self‐assembled peptides, supramolecular hydrogels, type 1 diabetes

## Abstract

Supramolecular peptide hydrogel has shown promising potential in vaccine development largely because of its ability to function both as antigen depot and immune adjuvant. Nap‐G^d^F^d^F^d^Y, a tetrapeptide hydrogel that has been previously reported to exhibit adjuvant effect, is inadvertently found to contain conserved peptide sequence for insulin, proinsulin, and glutamic acid decarboxylase, 3 major autoantigens for the autoimmune type 1 diabetes (T1D). At present, despite being managed clinically with insulin replacement therapy, T1D remains a major health threat with rapidly increasing incidences, especially in children and young adults, and antigen‐specific immune tolerance induction has been proposed as a feasible approach to prevent or delay T1D progression at an early stage. Here, it is reported that innoculation of Nap‐G^d^F^d^F^d^Y leads to complete protection of nonobese diabetic (NOD) mice from T1D development till the age of 36 weeks. Better maintenance of pancreatic islet morphology with minimal immune cell infiltration is also observed from mice exposed to Nap‐G^d^F^d^F^d^Y. This beneficial impact is mainly due to its facilitative role on enhancing peripheral T regulatory cell (Treg) population, shown as increased splenic Treg percentage, and function, demonstrated by maintenance of circulating TGF‐*β*1 level. Serum cytokine microarray data further implicate a “buffering” role of Nap‐G^d^F^d^F^d^Y on systemic inflammatory tone in NOD mice. Thus, with its versatility, applicability, and excellent potency, Nap‐G^d^F^d^F^d^Y is posited as a novel therapeutic intervention for T1D.

## Graphical abstract

A hydrogel of Nap‐GdFdFdY containing conserved sequence of autoantigens including insulin, proinsulin, and glutamic acid decarboxylase as a novel therapeutic intervention for the autoimmune type 1 diabetes. Better pancreatic islet morphology with minimal immune cell infiltration is observed from mice with hydrogel because of enhancing peripheral T regulatory cell population and maintenance of circulating TGF‐β1 level.

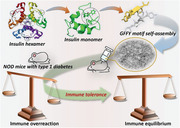

## 1 Introduction

Diabetes is a chronic systemic disorder characterized by deficiency of endogenous insulin and sustained hyperglycemia. Type 1 diabetes (T1D) is one subtype of diabetes that is caused by autoimmune attacks to self‐insulin‐producing *β*‐cells. It accounts for ≈10% of all cases of diabetes and the incidence of T1D is rising at 2–3% annually.^[^ ^1^ ^]^ Although onset of T1D can be observed in people of any age with nearly 50% of cases diagnosed in adults,^[^ ^2^ ^]^ the major population showing the greatest increase in T1D onset is children under the age of 15.^[^ ^3^ ^]^ Due to its relatively early onset and wide prevalence, T1D is the major cause of various health problems including cardiovascular diseases, kidney failure, infection, lower limb amputation, and blindness.

Despite extensive research effort, therapeutic approaches that can effectively reverse diabetes remain elusive. Antigen‐specific immune tolerance induction has been proposed as a possible option to ameliorate symptoms of autoimmune disorders, such as allergy,^[^ ^4^ ^]^ multiple sclerosis,^[^ ^5^ ^]^ and T1D.^[^ ^6^ ^]^ But for T1D, inoculation with single autoantigens including insulin,^[^ ^7^ ^]^ proinsulin,^[^ ^8^ ^]^ glutamic acid decarboxylase (GAD)^[^ ^9^ ^]^ has been associated with less than satisfactory clinical outcome.^[^ ^1^, ^9^, ^10^ ^]^ In order to improve potency and specificity, administration of immune dominant peptides has been adopted for immune tolerance induction, although the typically short circulating lifespan of peptides often renders this option lack of effectiveness in terms of offering immune protection.^[^ ^11^ ^]^ As a result, biomaterial‐based approaches such as engineering of peptide‐MHC (pMHC) nanomedicine,^[^ ^12^ ^]^ spatiotemporal delivery of peptide antigen via hydrogel/microparticle‐delivery system^[^ ^13^ ^]^ have been introduced to overcome hypersensitivity and lack of potency that are typically associated with in vivo delivery of protein/peptide drugs.^[^ ^14^ ^]^ Moreover, for T1D in particular, it has been reported that presence of more than two types of autoantigens are often found in patients with full onset of T1D, implicating a more complex aetiology of the disease.^[^ ^1^ ^]^

**Figure 1:**
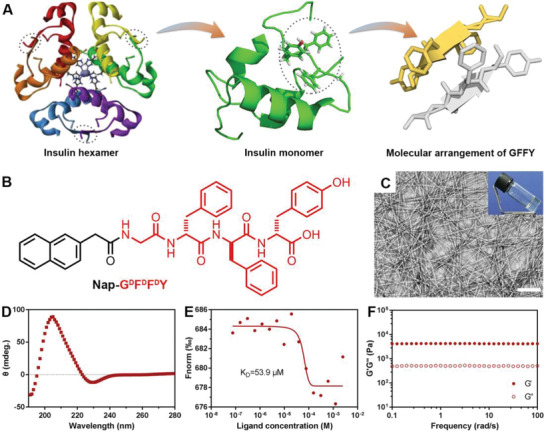
The insulin mimicking property of Nap‐G^d^F^d^F^d^Y hydrogel. The structural representation of A) insulin hexamer, insulin monomer, and self‐assembled Gly‐Phe‐Phe‐Tyr (GFFY) in insulin. The dotted areas indicate location of GFFY within insulin monomer and hexamer, in which GFFY is important for insulin receptor interaction and is essential for providing structural activity such as hexamer stabilization and dissociation. B) The chemical structure of self‐assembled peptide Nap‐G^d^F^d^F^d^Y. C) The optical image of hydrogel and representative image obtained by transmission electron microscopy (TEM). Scale bar = 100 nm. D) The circular dichroism spectrum of peptide in hydrogel of Nap‐G^d^F^d^F^d^Y. E) The fitting curve of microscale thermophoresis (MST) to calculate the *K*_D_ value of Nap‐G^d^F^d^F^d^Y to insulin receptor. F) The dynamic frequency sweep of hydrogel at the strain value of 1%.

Biomedical applications of supramolecular peptide‐based nanomaterials have been extensively reported, previously in the field of drug delivery and more recently, immune modulation.^[^ ^15^ ^]^ We have previously designed supramolecular hydrogels based on tetrapeptide Gly‐^d^Phe^d^‐Phe^d^‐Tyr^d^ (G^d^F^d^F^d^Y) and reported their stimulatory impact on T lymphocytes for cancer vaccine development.^[^ ^16^ ^]^ More importantly, in our efforts to elucidate the underlying mechanisms of actions of these hydrogels in immune modulation, we inadvertently discovered that T1D autoantigens proinsulin, insulin, and GAD all share peptide sequence of either GFFY or GFWY in both mouse and human (proinsulin; insulin B chain 23‐26; GAD65 506‐508; GAD67 514‐517).^[^ ^17^ ^]^ Moreover, it has been shown that GFFY is crucial both for insulin storage (stabilization of insulin hexamer) and insulin action (provides structural activity for insulin monomer dissociation and insulin receptor activity^[^ ^18^ ^]^ (**Figure** **1**A; Figure S1, Supporting Information). Recently reported by DiMeglio et al.,^[^ ^1^ ^]^ individuals with one single autoantibody often do not progress to establishing autoimmune diabetes, while presence of more than 2 autoantibodies was shown to have 84% chance of T1D development. As a result, considering that GFFY‐based hydrogels may potentially mimic multiple major autoantigens of T1D, in addition to their in vivo immune modulatory potential, we have investigated the impact of Nap‐G^d^F^d^F^d^Y^d^ (Figure 1B) for antigen‐specific immune tolerance induction against progression of autoimmune diabetes.

## 2 Results

### 2.1 Structural and Biological Characterization of Peptide Hydrogel/Formula

We first tested the gelation properties of Nap‐G^d^F^d^F^d^Y and controlled peptides (Nap‐GFFY, Nap‐^d^F^d^F, Nap‐G^d^F^d^A^d^Y, and Ac‐G^d^F^d^F^d^Y). After heating–cooling, Nap‐G^d^F^d^F^d^Y, Nap‐GFFY, and Nap‐^d^F^d^F could swiftly form transparent hydrogels (Figure 1C; Figure S3A,B, Supporting Information), but Nap‐G^d^F^d^A^d^Y and Ac‐G^d^F^d^F^d^Y formed solutions (Figure S3C,D, Supporting Information). The nanostructures of these hydrogels and solutions were subsequently observed by transmission electron microscopy (TEM). As shown in Figure 1C, the gel of Nap‐G^d^F^d^F^d^Y had straight nanofibers, which crosslinked to form a network. The diameter of its nanofiber was about 12 nm. The gel of Nap‐GFFY exhibited similar nanoscale morphology and nanometer size with the one of Nap‐G^d^F^d^F^d^Y (Figure S3E, Supporting Information). Nap‐^d^F^d^F formed thicker nanofibers with diameter of about 20 nm (Figure S3F, Supporting Information). However, Nap‐G^d^F^d^A^d^Y and Ac‐G^d^F^d^F^d^Y merely self‐assembled into short nanofibers or amorphous aggregation (Figure S3G,H, Supporting Information). Insulin mimicking requires a specific conformation of the self‐assembled peptide Nap‐G^d^F^d^F^d^Y for in vivo performance, indicating a secondary structure examination is required. We obtained the circular dichroism (CD) spectrum of peptide within the hydrogel at 37 °C. As shown in Figure 1D, peptide within the hydrogel exhibit an antiparallel *β*‐sheet conformation characterized by a groove observed at 192 nm and a peak shown near 205 nm, implicative of a reasonably good mimicking potential of the hydrogel to the spatial conformation of insulin B chain due to its molecular arrangement (Figure 1A). Indeed, when we tested for the binding kinetics of Nap‐G^d^F^d^F^d^Y peptide with human insulin receptor protein, the dissociation constant value (*K*_D_) was 5.387 × 10^−5^ (53.9 × 10^−6^ m), suggesting a moderate binding affinity to the insulin receptor and an insulin mimicking property of Nap‐G^d^F^d^F^d^Y (Figure 1E).^[^ ^19^ ^]^

Ulteriorly, the mechanical properties of hydrogels were assessed by rheological analysis at 37 °C. As shown in Figure 1F, the dynamic frequency sweep of Nap‐G^d^F^d^F^d^Y hydrogel exhibited the values of the storage modulus (*G*′) and the loss modulus (*G*″) had a feeblish dependence in the range of 0.1–100% of frequency. Combined with the observed ascendant *G*′ values (approximately ten times higher than *G*″ values), these results indicated that the hydrogel was a viscoelastic soft matter. Relatively equal rheological results implied the hydrogel of Nap‐GFFY possessed similar mechanical properties with the one of Nap‐G^d^F^d^F^d^Y (Figure S4, Supporting Information). The hydrogel of Nap‐^d^F^d^F showed increased dependency in the later stages of the sweep, meaning a stability change. The frequency sweep of Nap‐G^d^F^d^A^d^Y or Ac‐G^d^F^d^F^d^Y solutions exhibited dominant *G*″ values, indicating their good fluidity. Peptide release from hydrogel/solution were also assessed. As shown in Figure S5 in the Supporting Information, Nap‐G^d^F^d^F^d^Y and Nap‐GFFY hydrogels exhibited the longest retention time compared to the rest of formulas, only about 40% of peptides were released from the gel within 12 h of test. Nap‐^d^F^d^F released about 70%. As expected the nongel forming Nap‐G^d^F^d^A^d^Y and Ac‐G^d^F^d^F^d^Y were completely released in less than 6 h. For in vivo metabolism of hydrogels/formulas, we observed the retention time of different groups. As shown in Figure S6 in the Supporting Information, nongel forming Nap‐G^d^F^d^A^d^Y and Ac‐G^d^F^d^F^d^Y more rapidly cleared in vivo, indicated by loss of fluorescence signal. The fluorescence signal of Nap‐GFFY and Nap‐^d^F^d^F group lasted about 30 h. However, Nap‐G^d^F^d^F^d^Y hydrogel had considerable retention time in the subcutaneous tissue (about 36 h). These results indicated that the stabilities and the supramolecular assembly properties were sequence‐dependent, which to a great extent may affect biological activities of self‐assembling peptides.

### 2.2 Nap‐G^d^F^d^F^d^Y Prevented T1D Onset in nonobese diabetic (NOD) Mice

**Figure 2:**
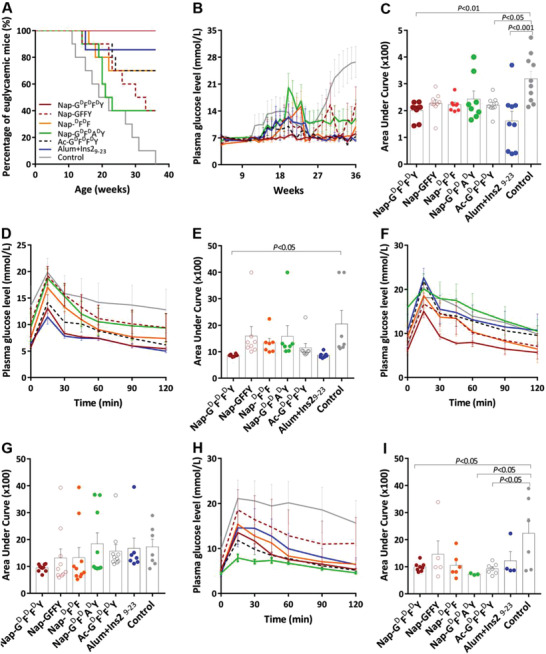
Effect of Nap‐G^d^F^d^F^d^Y on T1D progression and glucose tolerance in NOD mice. A) Incidence of type 1 diabetes onset. B) Weekly assessment and C) area under curve (AUC) analysis of random glucose levels. D) IPGTT result and E) AUC analysis at week 14. F) IPGTT result and G) AUC analysis at week 20. H) IPGTT result and I) AUC analysis at week 36. Data are presented as mean ± s.e.m., *n* = 7–10. Statistical significance was assessed using one‐way ANOVA with Bonferroni's post‐test. Nap‐G^d^F^d^F^d^Y: red solid line/red circle; Nap‐GFFY: red dotted line/red empty circle; Nap‐^d^F^d^F: orange solid line/orange circle; Nap‐G^d^F^d^A^d^Y: green solid line/green circle; Ac‐G^d^F^d^F^d^Y: black dotted line/black empty circle; Alum+Ins2_9‐23_: blue solid line/blue circle; Control: gray solid line/gray circle.

Female NOD mice were inoculated with different hydrogel formulations 3 weeks of age. PBS and aluminum hydroxide+Ins2_9‐23_ (Alum+Ins2_9‐23_) were used as negative and positive controls, respectively. Random blood glucose (RBG) levels were measured weekly and mice with RBG levels above 11.1 mmol L^−1^ for more than 3 consecutive weeks were considered “diabetic” in the present study. As shown in **Figure** **2**A, administration of Nap‐G^d^F^d^F^d^Y completely prevented the onset of T1D (Figure 2A, solid red line), significantly better than mice from all other groups. Administration of Alum+Ins2_9‐23_ led to 90% T1D prevention as expected, owning to the tolerance inducing property of Ins2_9‐23_.^[^ ^7^, ^20^ ^]^ However, 3 cases (out of 10) of adverse event (non‐T1D‐related deaths) were reported in the early stage of the study (Figure 2A, green solid line), consistent with previous publications reporting side effects of aluminum hydroxide.^[^ ^9a^ ^]^ Moreover, average body weight of mice from the Alum+Ins2_9‐23_ group was also shown to be significantly lower than the Nap‐G^d^F^d^F^d^Y, Ac‐G^d^F^d^F^d^Y, and Nap‐^d^F^d^F groups (Figure S8A,B, Supporting Information), reinstate the safety concerns of using aluminum hydroxide as adjuvant for immune tolerance induction.

Administration of Ac‐G^d^F^d^F^d^Y led to moderate level of T1D prevention (70% remained euglycemic; Figure 2A, black dotted line). This was expected despite the lack of gel‐forming capability of Ac‐G^d^F^d^F^d^Y, since antigenic impact of G^d^F^d^F^d^Y has been reported.^[^ ^17^ ^]^ In addition, Nap‐^d^F^d^F also elicited protective responses against T1D onset (70% remained euglycemic; Figure 2A, orange solid line), emphasizing structural importance of the two phenylalanines (FF). Indeed, the Gly‐Phe‐Phe‐Tyr (GFFY) sequence within the turn region of insulin B chain *β*‐sheet is conserved across a myriad of species in almost all vertebrates from mammals to rattlesnake, shark, and vulture,^[^ ^18b^ ^]^ and is involved not only in stabilizing insulin dimer and hexamer formation (Figure 1F–H) but also in insulin receptor interaction^[^ ^18a^ ^]^ (Figure S1, Supporting Information). In particular, the so‐called “aromatic triplet,” i.e., FFY, is critically required for providing distinct structure environment and association with insulin receptors as demonstrated by genetic manipulation and amino acid replacement experiments.^[^ ^18c^ ^]^ Moreover, mutations of either phenylalanines were found to be associated with glucose intolerance and hyperglycemia, emphasizing the importance of the two phenylalanines for insulin activity. This was further supported by our data showing significant T1D progression (60% T1D onset; Figure 2A, green solid line) of mice treated with Nap‐G^d^F^d^A^d^Y, as it lacks the two phenylalanines that are essential for insulin receptor interaction.

Poor tolerance induction was also observed from the Nap‐GFFY group (Figure 2A, red dotted line; 60% T1D onset). Previously, it has been suggested that the d‐isoform Nap‐G^d^F^d^F^d^Y was preferentially more stable in vivo than its l‐isoform counterpart Nap‐GFFY,^[^ ^16a^ ^]^ which may be attributable to its superior adjuvant effect during in vivo delivery of model antigen OVA.^[^ ^16b^ ^]^ In the present study, when both isoforms of Nap‐GFFY were used, the lack of stability and adjuvant potency of l‐GFFY resulted both in reduced antigenic and adjuvant impact, rendering the Nap‐GFFY formulation less effective for immune modulation in NOD mice.

Regarding plasma glucose concentrations (Figure 2B; Figure S5C–I, Supporting Information), it could be observed that random blood glucose levels of Nap‐G^d^F^d^F^d^Y (Figure S5C, Supporting Information), Ac‐G^d^F^d^F^d^Y (Figure S5D, Supporting Information), and Alum+Ins2_9‐23_ (Figure S5E, Supporting Information) were significantly lower than the Control group (Figure S5G, Supporting Information), consistent with T1D onset data (Figure 2A).

In order to assess glucose sensitivity, intraperitoneal glucose tolerance tests (IPGTTs) were also performed at 14 week (Figure 2D,E), 20 week (Figure 2F,G), and 36 week (Figure 2H,I). It was evident that as early as week 14, the Nap‐G^d^F^d^F^d^Y‐treated animals already exhibited better glucose tolerance than the Control group (AUC: 40 ± 1% over control, *p* < 0.05; Figure 2D,E). Despite no statistical significant differences at week 20 (Figure 2F,G), better glucose responsiveness of the Nap‐G^d^F^d^F^d^Y group was again recorded by week 36 (Nap‐G^d^F^d^F^d^Y AUC: 44 ± 3% over control, *p* < 0.05; Figure 2H,I). In addition, mice administrated with Ac‐G^d^F^d^F^d^Y were also more glucose sensitive than the control animals (AUC: 41 ± 3% over control, *p* < 0.05). However, rather unexpectedly, the Nap‐G^d^F^d^A^d^Y group also showed better glucose tolerance at 36 weeks compared to control (AUC: 34 ± 1% over control, *p* < 0.05). One explanation was that by week 36, more than half of mice from the Nap‐G^d^F^d^A^d^Y group had been withdrawn from the study due to severe hyperglycemia, and the very few that had survived were reasonably glucose tolerant (shown by individual plasma glucose levels presented in Figure S5H in the Supporting Information), and thus skewed the 36‐week IPGTT record for Nap‐G^d^F^d^A^d^Y.

### 2.3 Nap‐G^d^F^d^F^d^Y Preserved Pancreatic Islet Morphology while Prevented Immune Cell Infiltration

**Figure 3:**
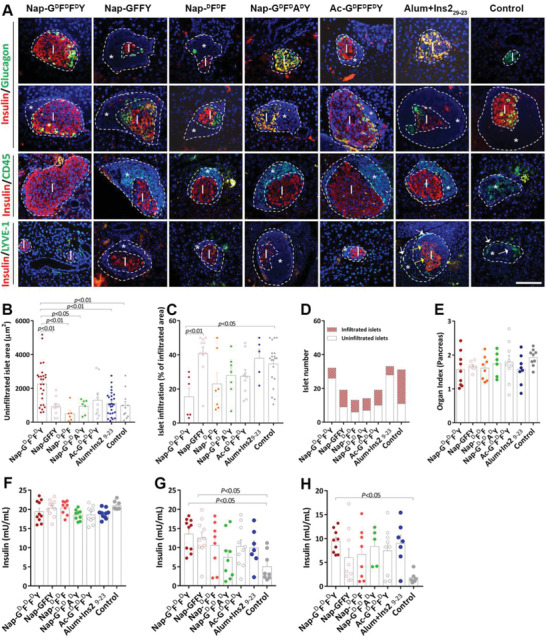
Effect of Nap‐G^d^F^d^F^d^Y on pancreatic islet morphology and immune cell infiltration. A) Immunostaining of mouse islets with insulin (red) and glucagon (green; top 2 rows); with insulin (red) and CD45 (green; third row); insulin (red) and LYVE‐1 (green; bottom row). B) Average area of unfiltrated islets, *n* = 6–28. C) Percentage of infiltrated islet area (infiltrated area over total islet area per islet), *n* = 5–20. D) Total islet number (red bar: number of infiltrated islets; white bar: number of uninfiltrated islets). E) Organ index of pancreas (ratio of pancreas weight over body weight). Plasma insulin levels of all mice from different treatment groups at F) week 14, G) week 20, and H) week 36, *n* = 7–10. Data presented as mean ± s.e.m. Statistical significance was assessed using one‐way ANOVA with Bonferroni's post‐test.

Pancreatic islet function is critically important to the maintenance of plasma glucose level and systemic metabolism. This is because the pancreatic *β*‐cells within the endocrine islets are the sole source of insulin production currently known in mammals. To further investigate the impact of Nap‐G^d^F^d^F^d^Y administration on diabetes progression, and more specifically, on pancreatic islet preservation, morphology of islets as well as islet immune cell infiltration were examined by immunofluorescence staining. As shown in **Figure** **3**A (top 2 rows), pancreas sections were co‐stained with insulin (red) and glucagon (green), the two major islet hormones responsible for maintaining plasma glucose and metabolic equilibrium. In general, islets of healthy mice are oval‐shaped and ranging between 50 and 200 µm in diameter. These islets are individual organoids mainly composed of a majority of *β*‐cells (≈80% of total cells), usually locate within the islet core area, *α*‐cells (≈10% of total cells) and some other islet cells towards the outer islet region.^[^ ^21^ ^]^ During T1D progression however, immune cells would attack the insulin‐producing *β*‐cells, a process that destroys islet *β*‐cells and its insulin producing ability. This process, also known as insulitis, is characterized by the presence of immune cells within islets and regarded as hallmark for diabetes progression.^[^ ^1^ ^]^

Similar to the plasma glucose record (Figure 2B; Figure S5C–I, Supporting Information), it was evident from Figure 3A (top 2 rows, islets are outlined by dotted lines, islet area is marked as “I” and infiltrated area is marked as “*”), islets from the Nap‐G^d^F^d^F^d^Y‐treated mice maintained relatively normal mouse islet morphology with minimal immune cell infiltration, showing significant larger uninfiltrated islet area compared to almost all other groups (2257 ± 236 µm^2^ for Nap‐G^d^F^d^F^d^Y, 230 ± 24% over Control, *p* < 0.01; 246 ± 26% over Nap‐GFFY, *p* < 0.01; 447 ± 47% over Nap‐^d^F^d^F, *p* < 0.01; 239 ± 25% over Nap‐G^d^F^d^A^d^Y, *p* < 0.05; 174 ± 18% over Ac‐G^d^F^d^F^d^Y, *p* > 0.05; 174 ± 18% over Alum+Ins2_9‐23_, *p* < 0.01; Figure 3B). In contrast, mice from the Nap‐GFFY and Control group exhibited substantial immune cell infiltration (average % of immune cell infiltration per islet: [Nap‐GFFY]: 38.5 ± 3.8% per islet, 248 ± 25% over Nap‐G^d^F^d^F^d^Y, *p* < 0.01; [Control]: 38.17 ± 2.7% per islet, 246 ± 18% over Nap‐G^d^F^d^F^d^Y, *p* < 0.05; Figure 3A–C). Moderate islet infiltration, albeit not statistical significant, was also detected from the Nap‐^d^F^d^F, Nap‐G^d^F^d^A^d^Y, Ac‐G^d^F^d^F^d^Y, and Alum+Ins2_9‐23_ groups (Figure 3A–C), demonstrative of *β*‐cell attrition. In addition, dedifferentiation of islet *β*‐cells, i.e., reverse differentiation of *β*‐cells back to their precursor *α*‐cells, was observed from Control (Figure 3A Control, upper panel), Nap‐G^d^F^d^A^d^Y (Figure 3A Nap‐G^d^F^d^A^d^Y, upper panel) and Alum+Ins2_9‐23_ (Figure 3A Alum+Ins2_9‐23_, upper panel), indicating a loss of *β*‐cell identity.^[^ ^22^ ^]^ This was further confirmed by plasma insulin results, where progressive reduction of systemic insulin was shown in Control (Figure 3F week 14; Figure 3G week 20; Figure 3H week 36).

To further examine the process of islet immune cell infiltration, pancreas sections were double‐labeled with insulin (Figure 3A third row; shown in red) and lymphocyte maker CD45 (Figure 3A third row; shown in green), or insulin and LYVE‐1 for lymphatic vessels (Figure 3A bottom row; insulin in red and LYVE‐1 in green). It could be seen that hardly any green fluorescence that corresponds to CD45 or LYVE‐1 could be detected from mouse islets of the Nap‐G^d^F^d^F^d^Y group (Figure 3B), again demonstrating minimal immune cell infiltration. As expected, islets from other treatment groups exhibited various degree of immune cell infiltration (Figure 3B–D) and majority of the infiltrated cells were of lymphatic origin since most were stained positive for CD45 (Figure 3A third row).

Consistently, percentage of uninfiltrated islets over total islets observed was also high for both Nap‐G^d^F^d^F^d^Y and Alum+Ins2_9‐23_ (81% and 85%, respectively; Figure 3D white bar). It is worth mentioning that although the ratio of uninfiltrated islet number over total islet number is high for Alum+Ins2_9‐23_, closer examination revealed that, for Alum+Ins2_9‐23_, the islets were smaller than those of Nap‐G^d^F^d^F^d^Y (shown in Figure 3B as uninfiltrated islet area, 208 ± 22% over Nap‐G^d^F^d^F^d^Y, *p* < 0.01). Besides, as mentioned earlier, more than half of islets observed from the Alum+Ins2_9‐23_‐treated mice exhibited significant *β*‐cell dedifferentiation (Figure 3A Alum+Ins2_9‐23_ upper panel, shown as loss of red fluorescence that corresponds to islet insulin expression and increased yellow fluorescence as a result of double‐labeling of both insulin (red) and glucagon (green)), both of which are indicators of worsened islet function. Note that no significant differences were detected in terms of organ index for pancreas (organ index is the ratio of weight of pancreas over body weight; Figure 3E). The pancreas is composed of 20% endocrine islets and 80% exocrine tissues mainly responsible for digestion. This result perhaps implies that preservation of islet morphology and function by Nap‐G^d^F^d^F^d^Y is selective for the self‐immunogenic insulin‐producing *β*‐cells, and therefore restricted to the endocrine pancreas in NOD mice. Interestingly though, only islets from the Alum+Ins2_9‐23_ and Control groups were seen to be surrounded by LYVE‐1‐positive lymphatic vessels (Figure 3A lower panel, shown in green, white arrows). For Alum+Ins2_9‐23_ in particular, fluorescence signals correspond to LYVE‐1 were even seen within islets (Figure 3A lower panel, yellow arrows). This lymphatic hyperplasia could be due to the potent adjuvant proinflammatory role of aluminum hydroxide.^[^ ^9a^ ^]^

To summarize, we found that Nap‐G^d^F^d^F^d^Y was superior in protecting pancreatic islets from immune cell infiltration, *β*‐cell dedifferentiation, and islet cell attrition, which consequently led to better prevention against onset of T1D.

### 2.4 Nap‐G^d^F^d^F^d^Y Prevented T1D Onset via Upregulation of Peripheral T Regulatory Cell (Treg) Population and Function

It has long been established that Tregs are critically important to the induction of immune tolerance.^[^ ^23^ ^]^ Indeed, intravenous transfusion of surrogate Tregs has been proposed as one of the potential therapeutic approaches against autoimmune disorders.^[^ ^1^ ^]^ More recently, pMHC engineering for modulation of peripheral Tregs was also introduced with promising potential,^[^ ^15b^ ^]^ although engineering of multiresponsive pMHC remains challenging. Nevertheless, considering the importance of peripheral Tregs for tolerance initiation, we investigated whether the preventive role of Nap‐G^d^F^d^F^d^Y treatment on T1D development was mediated by its potential impact on Tregs.

**Figure 4:**
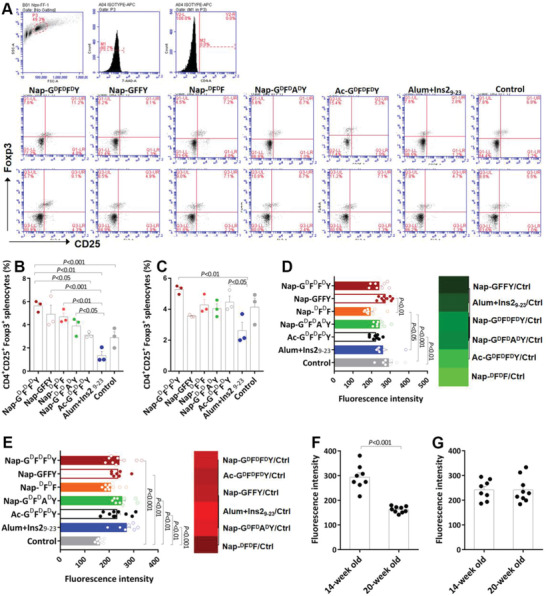
Effect of Nap‐G^d^F^d^F^d^Y on splenic Treg cell population and circulating TGF‐*β*1. A) Representative flow cytometry dot‐plots and gating information of splenic CD4^+^CD25^+^Foxp3^+^ Treg cells. Calculated percentage of CD4^+^CD25^+^Foxp3^+^ Tregs over total splenocytes of B) week 14 and C) week 20, *n* = 3. Mean fluorescence intensity of serum TGF‐*β*1 and microarray cluster map at D) week 14 and E) week 20, *n* = 7–10. Mean fluorescence intensity of serum TGF‐*β*1 of Control group at F) week 14 and G) week 20, *n* = 7–10. Data presented as mean ± s.e.m. Statistical significance was assessed using one‐way ANOVA with Bonferroni's post‐test.

As shown in **Figure** **4**A,B, as of week 14, the highest percentage of splenic CD4^+^CD25^+^Foxp3^+^ Tregs was detected from the Nap‐G^d^F^d^F^d^Y‐treated mice, significantly elevated than Control, Alum+Ins2_9‐23_, and Ac‐G^d^F^d^F^d^Y groups (181 ± 9%, 419 ± 21%, and 194 ± 10% over control, Alum+Ins2_9‐23_, and Ac‐G^d^F^d^F^d^Y, respectively, *p* < 0.05). Interestingly, lowest splenic Treg proportion was detected in Alum+Ins2_9‐23_ (Figure 4A,B), in contrast to its ameliorated T1D progression (Figure 2A). In fact, contradictory results regarding the effect of aluminum hydroxide for tolerance induction. Indeed, in earlier reports, repeated injection of Alum+GAD into lymph nodes has been shown to initiate immune protection against T1D development in clinical trials via activation of Th2‐prone immune responses.^[^ ^24^ ^]^ On the other hand, less than positive outcome was demonstrated in separate reports.^[^ ^9^ ^]^ The positive impact of Alum+Ins2_9‐23_ observed in the present study could be due to a better tolerance inducing impact of the Ins2_9‐23_ self‐antigen than the previously used GAD.^[^ ^7^, ^20^ ^]^ Furthermore, side effects remain as major safety concern regarding the use of adjuvants and we did also observe 3 cases of nondisease‐associated adverse events from the Alum+Ins2_9‐23_ group. As a result, although proinflammatory immune adjuvants have all been proposed as facilitative in tolerance induction against autoimmune disorders,^[^ ^15a^ ^]^ safety, and controversial therapeutic outcome have always remained as major concerns.

Compare to the nongel forming Ac‐G^d^F^d^F^d^Y, percentage of Treg cells from the Nap‐G^d^F^d^F^d^Y group was also significantly upregulated, emphasizing a dual, i.e., both antigenic and adjuvant, role of Nap‐G^d^F^d^F^d^Y hydrogel instead of Ac‐G^d^F^d^F^d^Y of which only antigenic effect was likely exerted. By week 20 (a period of time during which hyperglycemia have occurred in a significant proportion of untreated NOD mice, Figure 2A), percentage of splenic Tregs remained the highest in the Nap‐G^d^F^d^F^d^Y group though not statistically significant (Figure 4C), implicating a period‐specific action of Nap‐G^d^F^d^F^d^Y on peripheral Treg activation.

Regarding functional assessment of Tregs, we collected sera from mice of all groups for cytokine chip microarray. TGF‐*β*1 has been considered an important regulator of cell differentiation, proliferation, and survival. By releasing immune regulatory cytokines such as TGF‐*β*1, Tregs dampen the autoimmune attacks initiated by autoantigen recognizing autoreactive T cells. TGF‐*β*1 is also critical in inhibiting circulating autoreactive T cells that manage to escape the thymus T‐cell negative selection process. Moreover, TGF‐*β*1 knockout mice die early due to lack of immune tolerance and the resultant multifocal autoimmune organ inflammation.^[^ ^25^ ^]^ Contrary to Figure 4A,B, which showed significant elevation of splenic Treg population from the Nap‐G^d^F^d^F^d^Y group, we found that at week 14, serum TGF‐*β*1 levels of Nap‐G^d^F^d^F^d^Y demonstrated no difference from all other groups (Figure 4D; [Nap‐G^d^F^d^F^d^Y]: 82 ± 5% over control, *p* = 0.1357). Importantly though, by week 20, serum TGF‐*β*1 expression of the Control group dropped significantly, considerably lower than the Nap‐G^d^F^d^F^d^Y, Ac‐G^d^F^d^F^d^Y, and Alum‐Ins2_9‐23_ groups (Figure 4E; [Nap‐G^d^F^d^F^d^Y]: 149 ± 11% over control, *p* < 0.01; [Ac‐G^d^F^d^F^d^Y]: 142 ± 9% over control, *p* < 0.01; [Alum‐Ins2_9‐23_]: 167 ± 10% over control, *p* < 0.01). This reduction in TGF‐*β*1 level (55 ± 1% decrease, percentage over 14 week Control group, *p* < 0.001; Figure 4F) also coincided with exacerbated hyperglycemia and T1D progression in the Control group from week 14 to week 20 (Figure 2A). In contrast, for mice treated with Nap‐G^d^F^d^F^d^Y, change in serum TGF‐*β*1 was minimal (Figure 4G). Our data suggest that Nap‐G^d^F^d^F^d^Y may have facilitated tolerance induction against T1D by maintaining peripheral Treg population and function. The discrepancy observed regarding data between Treg flow cytometry and circulating TGF‐*β*1 concentration may imply a sequential activity of Nap‐G^d^F^d^F^d^Y, i.e., first augmenting splenic Treg population and subsequently maintaining TGF‐*β*1 production.

### 2.5 Effect of Nap‐G^d^F^d^F^d^Y Administration on Serum Cytokine Expression

**Figure 5:**
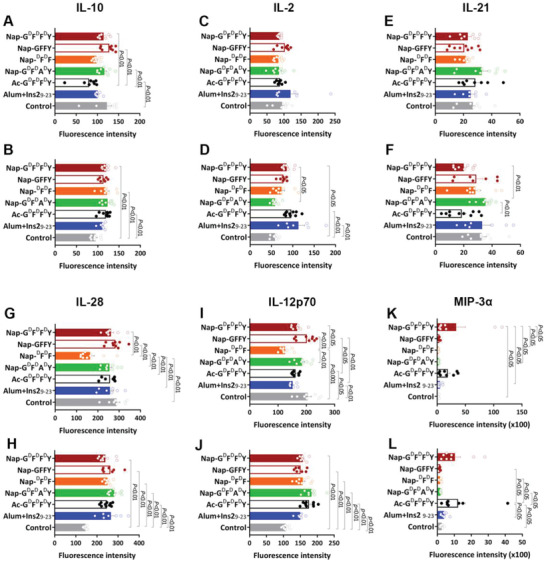
Effect of Nap‐G^d^F^d^F^d^Y on circulating cytokine expression. Mean fluorescence intensity of serum IL‐10 (A: week 14; B: week 20), IL‐2 (C: week 14; D: week 20), IL‐21 (E: week 14; F: week 20), IL‐28 (G: week 14; H: week 20), IL‐12p70 (I: week 14; J: week 20), and MIP‐3*α* (K: week 14; L: week 20). Data presented as mean ± s.e.m., *n* = 7–10. Statistical significance was assessed using one‐way ANOVA with Bonferroni's post‐test.

The positive role of Nap‐G^d^F^d^F^d^Y on tolerance induction could also be the result of its stimulatory impact on circulating IL‐10 at week 20 (**Figure** **5**A,B), which typically downregulates diabetogenic T cell activities ([Nap‐G^d^F^d^F^d^Y]: 126 ± 4% over control, *p* < 0.001; Figure 5B).^[^ ^26^ ^]^ However, it should be noted that similar to serum TGF‐*β*1 (Figure 4D,E), no difference between IL‐10 level of the Nap‐G^d^F^d^F^d^Y and Control groups was detected at week 14 ([Nap‐G^d^F^d^F^d^Y]: 93 ± 3% over control, *p* = 0.9691; Figure 5). Furthermore, expression of circulating IL‐2, a cytokine that is essential for the maintenance of Foxp3 expression,^[^ ^27^ ^]^ also exhibited similar pattern: statistical difference was only observable at week 20 but not week 14 ([Nap‐G^d^F^d^F^d^Y] week 20: 147 ± 7% over control, *p* < 0.05; [Nap‐G^d^F^d^F^d^Y] week 14: 92 ± 2% over Control, *p* = 0.9968; Figure 5E,F). The collective lack of statistical significance of multiple toleragenic cytokine expression at week 14 could be attributable to the transient and dynamic nature of cytokine production, which renders detection difficult and less sensitive.

At week 20, a period when more than 50% of mice from the Control group have established T1D phenotype, elevation of these cytokines is in line with observations published in human studies, reporting elevation of IL‐2, IL‐10, and IFN‐*γ* in healthy individuals when compared to T1D patients irrespective of glycemic management.^[^ ^28^ ^]^ Indeed, T‐cell and macrophage‐originated cytokines, such as IL‐2, IL‐10, and TGF‐*β*1, have all been implicated to exert impact locally on pancreatic *β*‐cell proliferation prior to immune cell infiltration,^[^ ^29^ ^]^ which could also be important in T1D progression and therapy development.

It is also worth noting that IL‐21, a major pathogenic proinflammatory cytokine in T1D^[^ ^30^ ^]^ was downregulated in the Nap‐G^d^F^d^F^d^Y group as compared to Control and Nap‐G^d^F^d^A^d^Y groups, although the differences were largely not statistically significant ([Nap‐G^d^F^d^F^d^Y] 14 week: 86 ± 10% over control, *p* = 0.9823; 70 ± 8% over Nap‐G^d^A^d^F^d^Y, *p* = 0.2648; [Nap‐G^d^F^d^F^d^Y] 20 week: 62 ± 7% over control, *p* = 0.1593; 57 ± 6% over Nap‐G^d^A^d^F^d^Y, *p* < 0.05; Figure 5E,F). Moreover, as shown in Figure 5C, the level of serum IL‐28 (also known as IFN‐*λ*) from Nap‐F^d^F^d^ group was significantly lower at week 14 ([Nap‐F^d^F^d^]: 57 ± 7% over control, *p* < 0.001), while no difference was detected among all other treatment groups. By week 20 however, the level of IL‐28 dropped considerably in the Control group (59 ± 1% over Nap‐G^d^F^d^F^d^Y, *p* < 0.001; Figure 5D). Previously, an antiviral role of IL‐28 in innate immunity modulation has been proposed,^[^ ^31^ ^]^ followed by more recent gene expression analyses revealing potential impact of IL‐28 on autoimmunity.^[^ ^31b^ ^]^ It has already been shown that protection of IL‐28 has been observed in allergic asthma via a Th1‐skewing action^[^ ^32^ ^]^ and autoimmune arthritis by reducing neutrophil recruitment to the affected joint region.^[^ ^33^ ^]^ As a result, it may be possible that the decrease of IL‐28 in the Control group observed at week 20 is indicative of diminished autoimmune protection, as suggested by increased incidence of T1D and decreased TGF‐*β*1 level in the Control group (Figures 2A and 4E). However, reduction of IL‐28 was observed from the Nap‐^d^F^d^F group at week 14, likely because Nap‐^d^F^d^F may interact with the insulin receptor and initiate immune responses differently from G^d^F^d^F^d^Y. Moreover, similar to our previous study reporting moderate adjuvant role of Nap‐G^d^F^d^F^d^Y on dendritic cell function,^[^ ^16b,d^ ^]^ elevation of IL‐12p70 (147 ± 4% over Control, *p* < 0.001, week 20; Figure 5I–J) and MIP‐3*α* (1316 ± 645% over Control, *p* < 0.05, week 14; Figure 5K,L) was observed from the Nap‐G^d^F^d^F^d^Y group in comparison to controls (Figure 5I–L), both of which are regarded as facilitative for the antigen presentation process, again supporting an adjuvant benefit of the Nap‐G^d^F^d^F^d^Y in NOD mice. Indeed, as we have reported previously, application of either Nap‐G^d^F^d^F^d^Y or Nap‐GFFY hydrogel can both elicited significant increase expression of CD40/CD80, two surface markers, on bone marrow derived dendritic cells (BMDCs), demonstrating positive impact of Nap‐G^d^F^d^F^d^Y on dendritic cell maturation and potential antigen presentation. Moreover, similar to data reported in the present study, level of TNF‐*α* in cell culture supernatant of BMDCs was also elevated following cell co‐culturing with Nap‐G^d^F^d^F^d^Y and Nap‐GFFY,^[^ ^16b^ ^]^ in line with cytokine expression profiling data in the present study.

Serum level of IL‐13, a cytokine that has been reported to both preserve pancreatic *β*‐cell viability^[^ ^34^ ^]^ and inhibit T effector cell attack,^[^ ^35^ ^]^ was also upregulated ([Nap‐G^d^F^d^F^d^Y]: 1316 ± 645% over Control, *p* < 0.05, week 20; Figure S9A,B, Supporting Information). No statistical significance was observed regarding the proinflammatory INF‐*γ*, TNF‐*α*, and IL‐6 (Figure S9C–H, Supporting Information) and the Th2‐biased IL‐4 and IL‐5 (Figure S9I–L, Supporting Information).

Considering that in vivo action of cytokines varies depending on local concentration and downstream signaling cascade,^[^ ^26^ ^]^ based on our results so far, we propose that the positive effect of Nap‐G^d^F^d^F^d^Y on T1D prevention is attributable to enhanced Treg function and a moderate and largely “buffering” role in maintaining systemic inflammatory tone.

### 2.6 Impact of Nap‐G^d^F^d^F^d^Y Treatment on Hyperglycemic Mice

**Figure 6:**
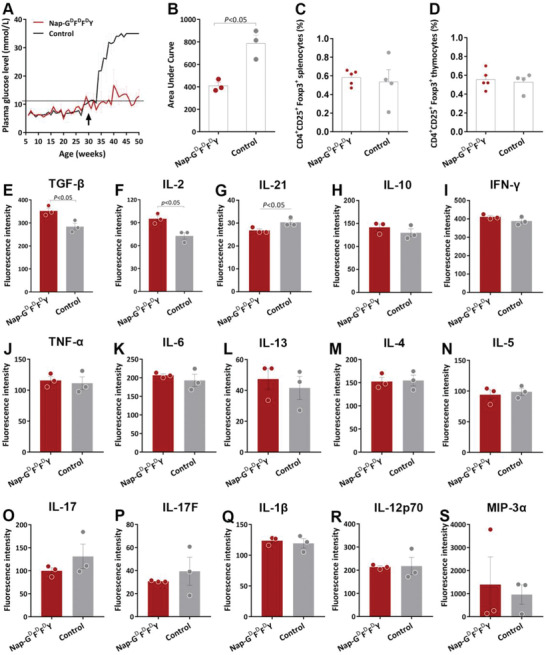
Effect of Nap‐G^d^F^d^F^d^Y in hyperglycemic NOD mice. A) Random plasma glucose levels of hyperglycemic NOD mice that had been treated with Nap‐G^d^F^d^F^d^Y (red solid line) and untreated Control (black solid line) at week 30 (black arrow). B) Area under curve analysis of random plasma glucose record for Nap‐G^d^F^d^F^d^Y (red circle) and Control (gray circle) groups. C) Percentage of CD4^+^CD25^+^Foxp3^+^ Tregs in spleen. D) Percentage of CD4^+^CD25^+^Foxp3^+^ Tregs in thymus. Average fluorescence intensity of serum expression of E) TGF‐*β*1, F) IL‐2, G) IL‐21, H) IL‐10, I) IFN‐*γ*, J) TNF‐*α*, K) IL‐6, L) IL‐13, M) IL‐4, N) IL‐5, O) IL‐17, P) IL‐17F, Q) IL‐1*β*, R) IL‐12p70, and S) MIP‐3*α*. Nap‐G^d^F^d^F^d^Y: red circle; Control: gray circle. Data presented as mean ± s.e.m., *n* = 3. Statistical significance was assessed using Student's *t*‐test.

To examine the potential impact of Nap‐G^d^F^d^F^d^Y on mice that were already hyperglycemic, i.e., with average plasma glucose level exceeds 11.1 mmol L^−1^, these mice were treated with Nap‐G^d^F^d^F^d^Y at week 30 and plasma glucose concentration was monitored weekly till 52 weeks of age. As shown in **Figure** **6**A,B, administration of Nap‐G^d^F^d^F^d^Y could not reverse hyperglycemia, although the average plasma glucose level was significantly lower in Nap‐G^d^F^d^F^d^Y‐treated mice than those from the untreated Control group (AUC: 52 ± 4% over control, *p* < 0.05; Figure 6A,B). This effect did not appear to be mediated by Treg‐dependent induction of immune tolerance since no difference could be observed from CD4^+^CD25^+^Foxp3^+^ Tregs from spleen (Figure 6C) and the thymus (Figure 6D). Although it could be because this is technically difficult due to the already low Treg percentage in hyperglycemic mice as well as the small sample size of this experiment. In addition, significant elevation of serum TGF‐*β*1 (Figure 6E) and IL‐2 (Figure 6F) levels was observed from the Nap‐G^d^F^d^F^d^Y‐treated animals in comparison to the controls ([TGF‐*β*1]: 124 ± 120% over control, *p* < 0.01; [IL‐2]: 132 ± 115% over control, *p* < 0.05). It has long been established that administration of low‐dose IL‐2 is beneficial for tolerance induction in T1D,^[^ ^36^ ^]^ and inhibition of T‐cell TGF‐*β*1 signaling would lead to development of autoimmune phenotype.^[^ ^25b^ ^]^ As a result, considering that both TGF‐*β*1 and IL‐2 are essential for the development and function of Treg and immune tolerance, our observation supports a facilitative role of Nap‐G^d^F^d^F^d^Y in Treg function during hyperglycemia.

In addition, IL‐21 level was lower in the Nap‐G^d^F^d^F^d^Y group, which was expected considering the comparatively better glycemic management of the Nap‐G^d^F^d^F^d^Y animals (87 ± 81% over control, *p* < 0.01; Figure 6G). No statistical significance was detected regarding serum levels of anti‐inflammatory IL‐10 (Figure 6H), IL‐4 (Figure 6M), IL‐13 (Figure 6L), proinflammatory cytokines IFN‐*γ* (Figure 6I), TNF‐*α* (Figure 6J), IL‐6 (Figure 6K), Th2‐biased IL‐5 (Figure 6N), Th17‐related IL‐17 (Figure 6O), IL‐17F (Figure 6P), and monocyte‐associated cytokines IL‐12p70 (Figure 6R), MIP‐3*α* (Figure 6S), implicating no major effect of Nap‐G^d^F^d^F^d^Y on Th1/Th2/Th17 modulation in hyperglycemic mice.

**Figure 7:**
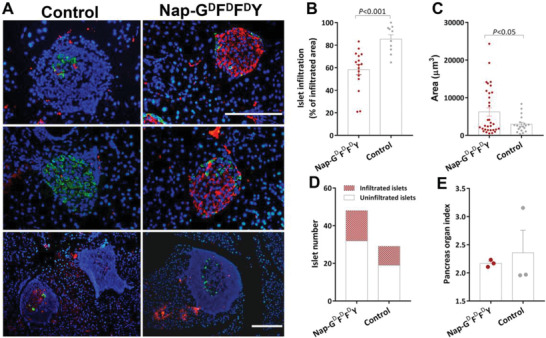
Effect of Nap‐G^d^F^d^F^d^Y on islet morphology and immune cell infiltration in hyperlycemia NOD mice. A) Immunofluorescence staining of insulin (red) and glucagon (green) using pancreas sections from hyperglycemic mice treated with Nap‐G^d^F^d^F^d^Y or untreated Control. B) Average percentage of infiltrated islet area (infiltrated area over total islet area per islet), *n* = 10–16. C) Average area of unfiltrated islets, 19‐32. D) Total islet number (red bar: number of infiltrated islets; white bar: number of uninfiltrated islets). E) Organ index of pancreas (ratio of pancreas weight over body weight), *n* = 3. Data are presented as mean ± s.e.m. Statistical significance was assessed by Student's *t*‐test.

In line with the plasma glucose data, morphological examination of pancreatic islets revealed comparatively less immune cell infiltration (68 ± 5% over control, *p* < 0.001; **Figure** **7**A,B), enlarged islet area (212 ± 38% over control, *p* < 0.05; Figure 7C) and increased number of islets (Figure 7D) from mice administrated with Nap‐G^d^F^d^F^d^Y. In addition to islet immune cell infiltration, insulin‐producing *β*‐cell dedifferentiation was also more frequently observed from pancreata of the control animals (Figure 7A), which also contribute to the continuous worsening of glycemic control of these animals (Figure 6A). However, significant islet infiltration was also present in the Nap‐G^d^F^d^F^d^Y group, in line with an ameliorative but not reversible benefit of Nap‐G^d^F^d^F^d^Y against hyperglycemia. The average pancreas organ index remains relatively indifferent (Figure 7E), although for the Control group individual values of pancreas weight varied significantly, which could be the result of advanced progression of hyperglycemia.

## 3 Conclusion

Supramolecular peptide hydrogel has, in the past 5 years, been extensively investigated as immune adjuvant to improve efficacy of antigen presentation. By using peptide hydrogel, which functions both as antigen depot and immune adjuvant, it eliminates the likely protein‐induced hypersensitivity in vivo and the need for additional adjuvant^[^ ^15^, ^37^ ^]^ We have previously reported moderate adjuvant impact of Nap‐G^d^F^d^F^d^Y for OVA‐induced immune responses.^[^ ^16b,d^ ^]^ Because the peptide sequence GFFY is conserved in multiple self‐antigens of autoimmune diabetes, we now have assessed its potential role in preventing onset of T1D in NOD mice. It is worth noting that majority of the current antidiabetic intervention approaches have been mainly targeting people who are already hyperglycemic. In order to achieve the widest coverage and impact, prevention aiming at high risk individuals should start as early as possible. As a result, we investigated the possible role of early administration of Nap‐G^d^F^d^F^d^Y against T1D progression by treating NOD mice well before the onset of hyperglycemia at 3 weeks of age. Therapeutic potential of Nap‐G^d^F^d^F^d^Y in mice that are already hyperglycemic was also evaluated.

We observed complete prevention of T1D onset till the age of week 36 by Nap‐G^d^F^d^F^d^Y, which was significantly better than the nongel‐forming Ac‐G^d^F^d^F^d^Y and the conventional aluminum hydroxide and self‐antigen (Ins2_9‐23_) combination (Alum+Ins2_9‐23_ group). Pancreatic islet *β*‐cell attrition, dedifferentiation, and immune cell infiltration were significantly alleviated in the Nap‐G^d^F^d^F^d^Y‐treated group, as a result of enhanced peripheral Treg cell proliferation and function as well as maintenance of circulating cytokine equilibrium. To summarize, our data demonstrate induction of immune tolerance by an insulin‐inspired Nap‐G^d^F^d^F^d^Y supramolecular hydrogel via its facilitative impact on peripheral Tregs and its capacity to reset systemic immunological tone. The preventive effect of Nap‐G^d^F^d^F^d^Y also implicates the possibility of implementing hydrogel‐based immune modulation at a much early stage prior to T1D onset, warranting it the possibility as a broad and more impactful contribution to the global effort in battling diabetes.

## 4 Experimental Section

##### Materials

2‐Cl‐trityl chloride resin (1.1 mmol g^−1^) was obtained from Nankai University resin Co., Ltd. Fmoc‐amino acids and *o*‐benzotriazol‐1‐yl‐*N*,*N*,*N*′,*N*′‐tetramethyluronium hexafluorophosphate (HBTU) were acquired from GL Biochem (Shanghai). Hydogels were prepared and respective endotoxin levels of formula were tested as previously reported.^[^ ^23^, ^24^ ^]^ Ins2_9‐23_ peptide (SHLVEALYLVCGERGR; endotoxins <1 EU mg^−1^) was supplied by Bankpeptide (Hefei, China). The BD Pharmingen FoxP3 staining kit was from (BD Biosciences, Beijing, China). Aluminum hydroxide was purchased from Sigma‐Aldrich (Shanghai, China). Antibodies used for flow cytometry analysis were purchased from eBioscience (CA, USA) and antibodies used for immunofluorescence staining were obtained from Abcam (Shanghai, China). Cytokine chip microarray was performed by Beijing Genomics Institute (Beijing, China). Recombinant Human Insulin R/CD220 (aa 28‐944) Protein was ordered from R&D Systems (Shanghai, China). Mouse Insulin EZRMI‐13 ELISA kit was from Millipore (Merck, UK). Female NOD/ShiLtJNju mice were supplied by Model Animal Research Center of Nanjing University. All animal procedures were approved by the Centre of Tianjin Animal Experiment Ethics Committee. The accreditation number of the laboratory is SYXK(Jin) 2019‐0003 promulgated by Tianjin Science and Technology Commission.

##### Peptide Synthesis and Hydrogel/Formula Characterization

All peptides in the study were synthesized by standard solid‐phase peptide synthesis (SPPS), and purified by high performance liquid chromatography (HPLC). Hydrogels/formulas were prepared by heating–cooling method. Negative staining technique was used to observe the nanostructures of materials by using TEM (HITACHI HT7700 Exalens). CD spectrum were measured by a BioLogic (MOS‐450) system. Rheology test was carried out on an AR 2000ex system (TA instrument). The release behaviors of peptides was analyzed by liquid chromatograph (LC2020). Microscale thermophoresis and analysis of binding force was performed by using the Monolith NT.115 system (NanoTemper Technologies, Germany). All samples were tested at the temperature of 37 °C.

##### Analysis of In Vivo Retention of Hydrogel/Formula

8‐weeks old female NOD mice were subcutaneously injected with a volume of 50 µL Cy5.5‐labeled peptide hydrogels/formulas in inguinal region. In vivo peptide retention was observed every 6 h at 640 nm excitation wavelength via Cri Maestro In‐vivo imaging System (Xenogen, IVIS Lumina II).

##### T1D Prevention Study

Female NOD mice (3 weeks of age) were immunized subcutaneously under the front left leg with Nap‐G^d^F^d^F^d^Y, Nap‐GFFY, Nap‐G^d^F^d^A^d^Y, Nap‐^d^F^d^F, Ac‐G^d^F^d^F^d^Y (1 mg mL^−1^, 50–70 µL), and Alum+Ins2_9‐23_ (50 µg) and endotoxin‐free PBS once a week for 5 consecutive weeks (from 3‐weeks old to 7‐weeks old) and maintained in specific pathogen free facilities for up to 36 or 52 weeks (*n* = 3–10 per group). Random plasma glucose level and body weight of all mice were noted weekly from week 8 till the end of experiment with an OneTouch Ultra blood glucose meter (Lifescan Inc., Milpitas, CA, USA). Mice were classified as diabetic if random plasma glucose level exceeded 11.1 mmol L^−1^ for 3 consecutive weeks. IPGTT was performed in all treatment groups at week 14, week 20, and week 36. Serum samples were obtained from all mice at the age of week 14, week 20, and week 36 for cytokine chip microarray, which was performed by the Beijing Genomics Institute. Serum insulin level was determined in 10 µL serum samples of each animal using a mouse insulin ELISA kit according to the manufacture's instruction.

##### Lymphocyte Purification from Thymus and Spleen

NOD mice were sacrificed and submerged in 75% ethanol for several minutes before dissection under aseptic condition. Thymus or spleen was extracted and grinded through a 70 µm BD cell strainer in lymphocyte extract solution and the lymphocytes were isolated by gradient purification (centrifugation at 800 × *g* for 30 min). Red blood cell lysis buffer was then added for 2 min before being washed with PBS. For Treg labeling, a BD Pharmingen FoxP3 staining kit was used according to the manufacture's instruction. Briefly, cells were first exposed to antimouse CD4 and antimouse CD25 antibodies and then treated with BD permeabilization buffer at 4 ℃ overnight. Antimouse Foxp3 antibodies were added and the cells were incubated for a further 30 min and analyzed immediately by BD Accuri C6 flow cytometry (BD Biosciences, San Jose, CA, USA).

##### Immunofluorescence Staining

Immunofluorescence staining was performed following procedures that were previously detailed.^[^ ^18b,c^ ^]^ Pancreas were extracted and paraffin‐embedded before being sectioned (5 µm width) onto microscopic slides. For immunofluorescence staining, antigen retrieval was performed followed by exposure to primary antibodies at 4 ℃ overnight. The pancreas sections were then washed with PBS and staining with secondary antibody for 1–2 h at room temperature and examined under a fluorescence microscope (Olympus BX3‐25ND25, Japan).

##### Statistics Analysis

All data were presented as means ± standard error of means (s.e.m.). Statistical analysis was performed using GraphPad Prism Software (CA, USA). For comparison between 2 groups, paired Student's *t*‐test was performed. For multiple group (3 groups and above) comparison, one‐way ANOVA analysis was employed with Bonferroni's post‐test. Group difference was considered statistically significant when a *p* value was below 0.05.

## Conflict of Interest

The authors declare no conflict of interest.

## Supporting information



Supporting Information

## Data Availability

The data that support the findings of this study are available from the corresponding author upon reasonable request.
