# Supplementary material for: An Insulin‐Inspired Supramolecular Hydrogel for Prevention of Type 1 Diabetes
Source: Adv Sci (Weinh). 2021 Apr 9;8(10):2003599. doi: 10.1002/advs.202003599 (PMC8132061; doi:10.1002/advs.202003599)

© 2021 Wiley-VCH GmbH

ADVANCED SCIENCE
Open Access

Supporting Information

for *Adv. Sci.*, DOI: 10.1002/advs.202003599

An Insulin-inspired Supramolecular Hydrogel for Prevention of Type 1 Diabetes

Mohan Liu[#], Zhongyan Wang[#], Dandan Feng, Yuna Shang, Xinxin Li, Jianfeng Liu, Chen Li and Zhimou Yang**

Electron Supplementary Information

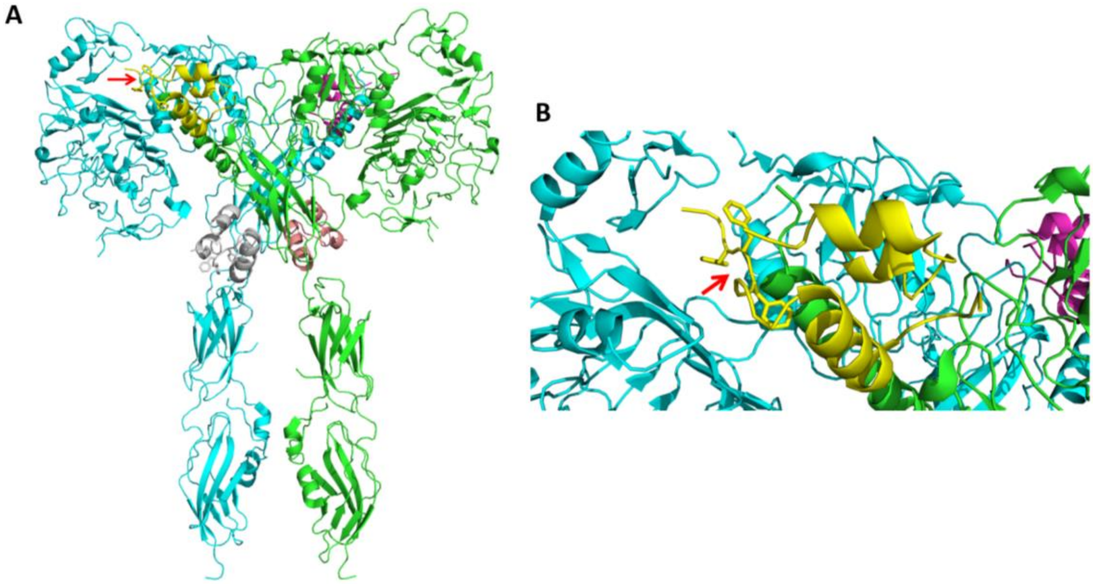

Supplementary Figure S1. Structural representation of interaction between insulin and insulin receptor. (A) Overview structure of insulin-insulin receptor complex. Red arrow indicates the GFFY sequence within the turn strand of β-sheet region within the insulin B chain. (B) Zoomed view of insulin-insulin receptor complex where GFFY (red arrow) interact with insulin receptor.

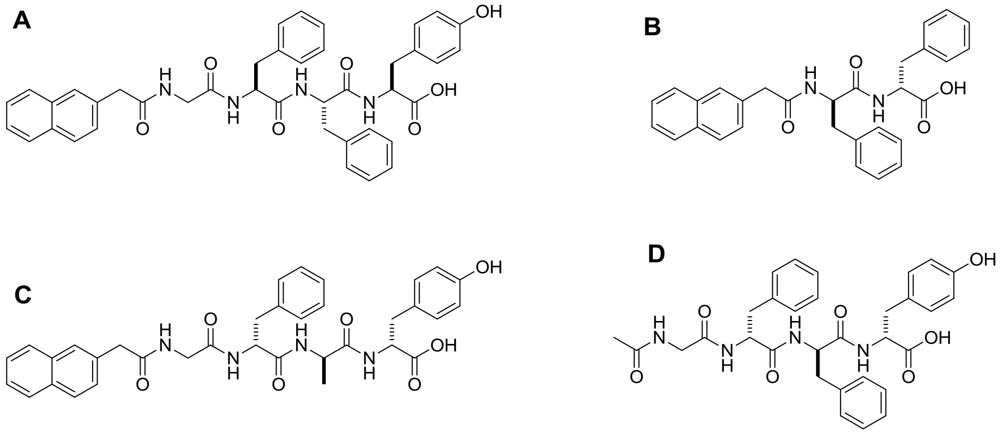

Supplementary Figure S2. The chemical structures of (A) Nap-GFFY, (B) Nap-^{D}F^{D}F, (C) Nap-G^{D}F^{D}A^{D}Y and (D) Ac-G^{D}F^{D}F^{D}Y, respectively.

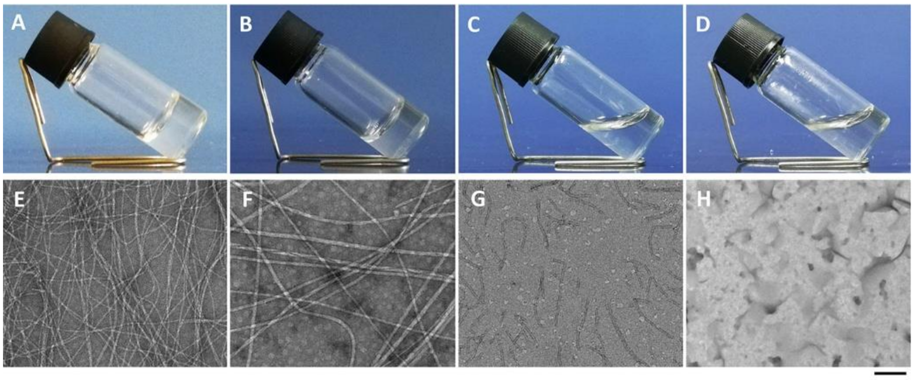

Supplementary Figure S3. The optical images of (A) Nap-GFFY, (B) Nap-^{D}F^{D}F, (C) Nap-G^{D}F^{D}A^{D}Y and (D) Ac-G^{D}F^{D}F^{D}Y and their corresponding nanostructures examined by TEM. Representative TEM images of (E) Nap-GFFY, (F) Nap-^{D}F^{D}F, (G) Nap-G^{D}F^{D}A^{D}Y and (H) Ac-G^{D}F^{D}F^{D}Y, scale bar = 100 nm.

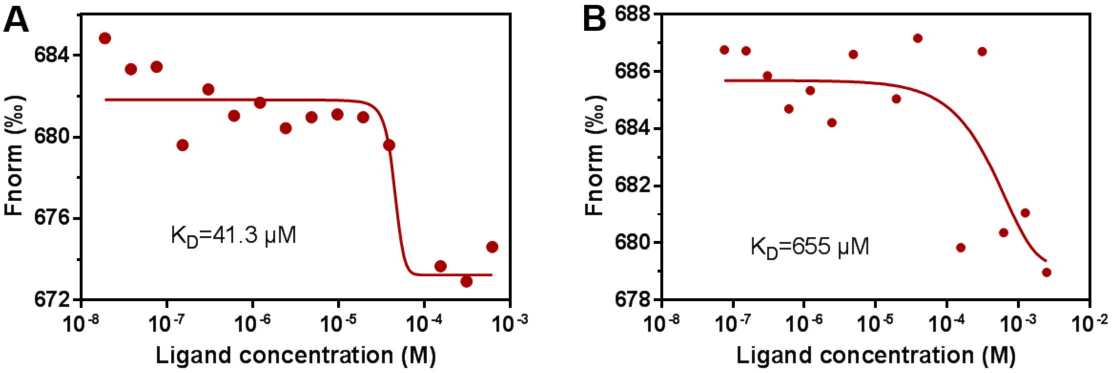

Supplementary Figure S4. The fitting curve of microscale thermophoresis (MST) to calculate the K_D value of (A) Nap-GFFY and (B) Nap-G^{D}F^{D}A^{D}Y to insulin receptor.

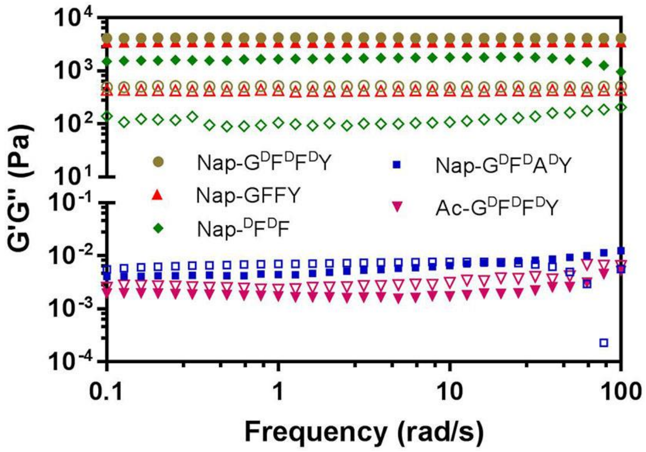

Supplementary Figure S5. The mechanical properties of the hydrogels of Nap-G^{D}F^{D}F^{D}Y, Nap-GFFY and Nap-^{D}F^{D}F, and the solution of Nap-G^{D}F^{D}A^{D}Y and Ac-G^{D}F^{D}F^{D}Y were analyzed by rheology.

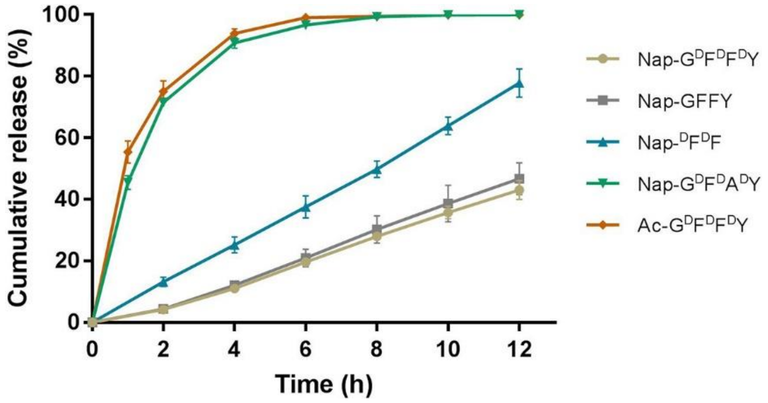

Supplementary Figure S6. The *in vitro* cumulative release curve of Nap-G^{D}F^{D}F^{D}Y, Nap-GFFY and Nap-^{D}F^{D}F from their hydrogel, and Nap-G^{D}F^{D}A^{D}Y and (H) Ac-G^{D}F^{D}F^{D}Y from their solution.

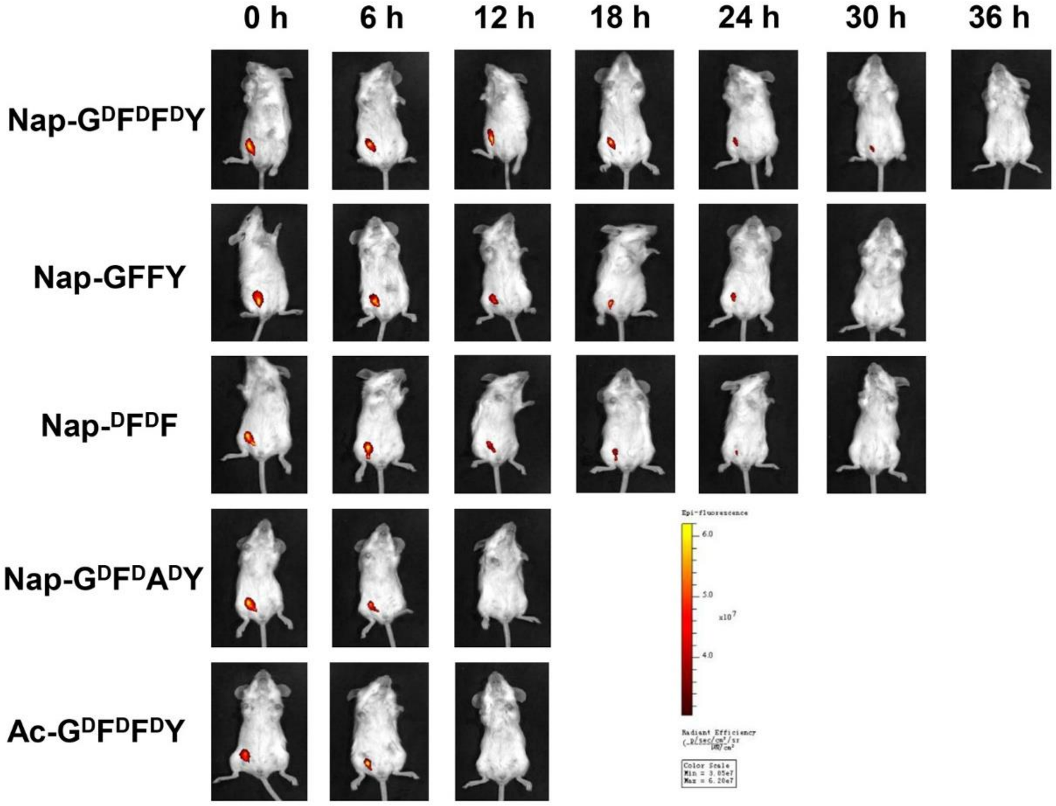

Supplementary Figure S7. *In vivo* retention of different peptide hyrogels/formulas.

Supplementary Figure S8 Average body weight and individual random blood glucose levels of mice from all treatment groups. (A-B) Weekly record **(A)** and AUC analysis **(B)** of mouse body weight. **(C-I)** Weekly plasma glucose level of individual mouse of Nap-G^{D}F^{D}F^{D}Y **(C)**, Ac-G^{D}F^{D}F^{D}Y **(D)**, Alum+Ins2$_{9\text{-}23}$ **(E)**, Nap-^{D}F^{D}F **(F)**, Control **(G)**, Nap-G^{D}F^{D}A^{D}Y **(H)** and Nap-G^{L}F^{L}F^{L}Y **(I)**. Data are presented as mean±s.e.m. Statistical significance was assessed using one-way ANOVA with Bonferroni's post-test. Nap-G^{D}F^{D}F^{D}Y: red solid line/red circle; Nap-G^{L}F^{L}F^{L}Y: red dotted line/red empty circle; Nap-^{D}F^{D}F: orange solid line/orange circle; Nap-G^{D}F^{D}A^{D}Y: green solid line/green circle; Ac-G^{D}F^{D}F^{D}Y: black dotted line/black empty circle; Alum+Ins2$_{9\text{-}23}$: blue solid line/blue circle; Control: grey solid line/grey circle.

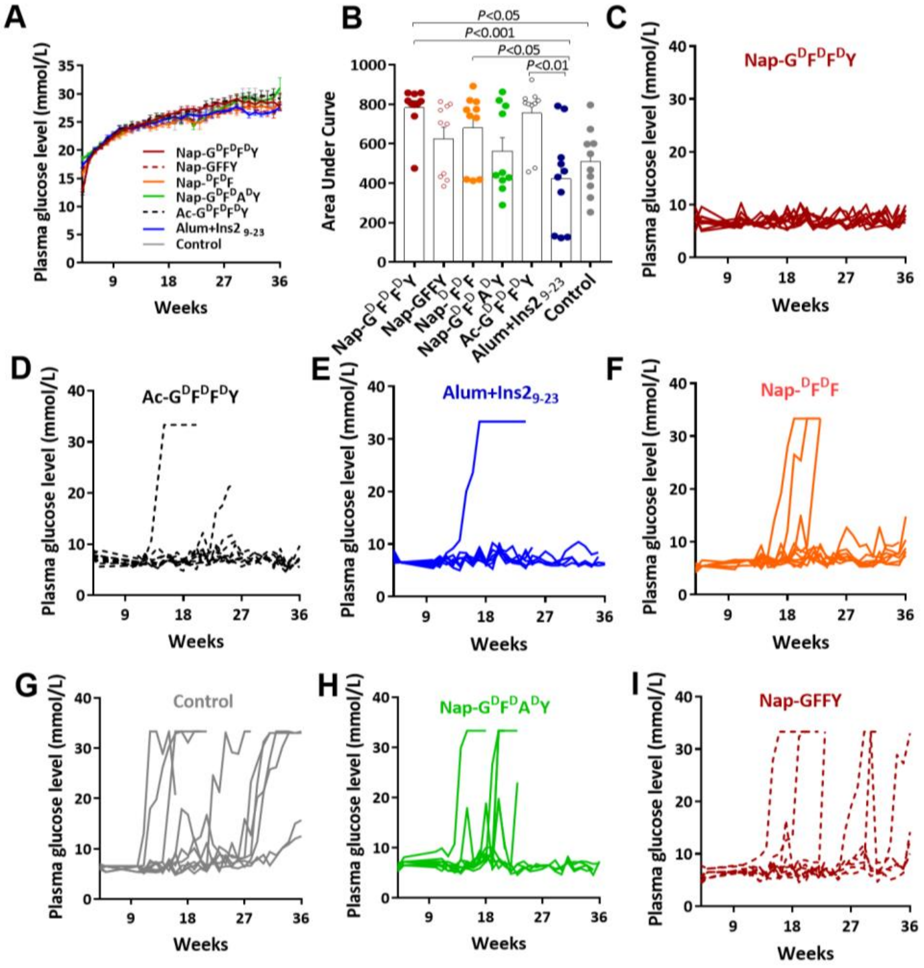

Supplementary Figure S9 Serum cytokine expression levels of mice from all treatment groups. Mean fluorescence intensity of serum IL-13 (**[A]**: week 14; **[B]**: week 20), TNFα (**[C]**: week 14; **[D]**: week 20), INF-γ (**[E]**: week 14; **[F]**: week 20), IL-6 (**[G]**: week 14; **[H]**: week 20), IL-4 (**[I]**:week 14; **[J]**: week 20), IL-5 (**[K]**: week 14; **[L]**: week 20), IL-17 (**[M]**: week 14, **[N]**: week 20), IL-17F (**[O]**: week 14, **[P]**: week 20), IL-1β (**[Q]**: week 14, **[R]**: week 20). Data presented as mean±s.e.m. Statistical significance was assessed using one-way ANOVA with Bonferroni's post-test.

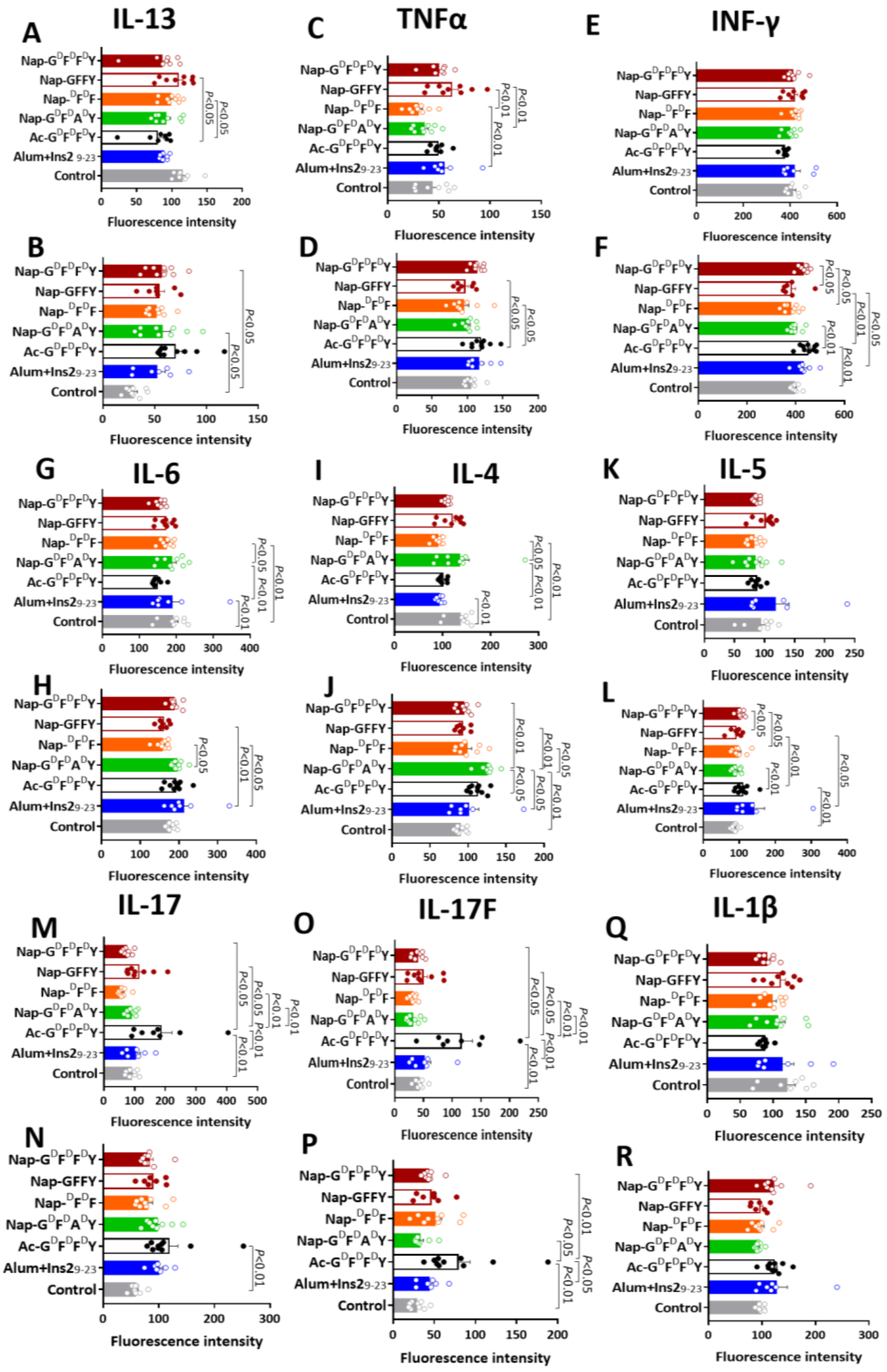

Supplement: Supplementary file 1 — Supporting Information [file ADVS-8-2003599-s001.pdf]
